# Exon Exchange Approach to Repair Duchenne Dystrophin Transcripts

**DOI:** 10.1371/journal.pone.0010894

**Published:** 2010-05-28

**Authors:** Stéphanie Lorain, Cécile Peccate, Maëva Le Hir, Luis Garcia

**Affiliations:** Université Pierre et Marie Curie (UPMC UMR S 974)-Institut National de la Santé et de la Recherche Médicale (Inserm U974)-Centre National de la Recherche Scientifique (CNRS UMR 7215)-Institut de Myologie, Paris, France; University of Florida, United States of America

## Abstract

**Background:**

Trans-splicing strategies for mRNA repair involve engineered transcripts designed to anneal target mRNAs in order to interfere with their natural splicing, giving rise to mRNA chimeras where endogenous mutated exons have been replaced by exogenous replacement sequences. A number of trans-splicing molecules have already been proposed for replacing either the 5′ or the 3′ part of transcripts to be repaired. Here, we show the feasibility of RNA surgery by using a double trans-splicing approach allowing the specific substitution of a given mutated exon.

**Methodology/Principal Findings:**

As a target we used a minigene encoding a fragment of the *mdx* dystrophin gene enclosing the mutated exon (exon 23). This minigene was cotransfected with a variety of exon exchange constructions, differing in their annealing domains. We obtained accurate and efficient replacement of exon 23 in the mRNA target. Adding up a downstream intronic splice enhancer DISE in the exon exchange molecule enhanced drastically its efficiency up to 25–45% of repair depending on the construction in use.

**Conclusions/Significance:**

These results demonstrate the possibility to fix up mutated exons, refurbish deleted exons and introduce protein motifs, while keeping natural untranslated sequences, which are essential for mRNA stability and translation regulation. Conversely to the well-known exon skipping, exon exchange has the advantage to be compatible with almost any type of mutations and more generally to a wide range of genetic conditions. In particular, it allows addressing disorders caused by dominant mutations.

## Introduction

Mutations in the dystrophin gene cause Duchenne muscular dystrophy (DMD), the most common severe childhood muscular pathology. Recently, exon skipping strategies have proven to be efficacious in restoring functional dystrophin expression in models of muscular dystrophy including the *mdx* mouse, the GRMD dog and muscle stem cells from DMD patients and in DMD patients by local intramuscular injection [1]–[6]. Indeed, the modular structure of the dystrophin, with its central rod-domain made of 24 spectrin-like repeats, tolerates large internal deletions [7]–[8]. However, exon skipping strategies only concern patients for whom forced splicing would generate a shorter but still functional protein. Many pathological situations escape this prerequisite. Fortunately, RNA repair strategies based on trans-splicing approaches could overcome this problem. Trans-splicing approaches or Spliceosome-mediated RNA trans-splicing (SMaRT; Intronn proprietary technology) [9] use RNA molecules that interfere with the natural splicing of targeted mRNAs in order to repair mutated gene products. Mostly, trans-splicing studies have developed therapeutic RNAs replacing the 3′ part of the transcript to be repaired. They have been applied to correct a number of mutations using minigenes or endogenous transcripts in genetic disease context like hemophilia A [10], spinal muscular atrophy [11], X-linked immunodeficiency [12] and cystic fibrosis where the widespread mutation CFTRΔF508 was replaced efficiently *in vivo* by the normal sequence via a trans-splicing reaction [13]. On the other hand, only a few attempts for 5′ replacement have been reported to be successfull on minigenes [14]–[15]. Conversely to conventional gene therapy, trans-splicing approaches could address disorders caused by dominant mutations, while preserving levels and tissue specificity.

Exon exchange using double trans-splicing at both sides of the targeted exon has never been achieved but its interest has already been underlined [16]–[18]. It would have the further advantages of minimizing exogenous material as well as preserving full regulatory elements potentially present in 5′ and/or 3′ untranslated domains of the rescued mRNA.

The study presented here relates the optimization of exon exchange (ExChange) constructs designed for rescuing mutated mRNAs from very large genes such as the dystrophin gene. The murine model for dystrophin mutations, the *mdx* mouse, carries a nonsense mutation in exon 23 of the dystrophin gene [19]. In order to test the proof of concept that double trans-splicing was feasible, we designed a minigene made of the murine dystrophin genomic fragment containing the *mdx* mutation; beginning at exon 22 and ending after the exon 24 under the control of a constitutive promoter. This minigene was cotransfected with a variety of exon exchange constructions, differing in their annealing domains. We show here for the first time that ExChange is possible. Indeed, we show accurate and efficient replacement of exon 23 in the target mRNA. Adding up a downstream intronic splice enhancer DISE in the ExChange molecule improved its efficiency up to 25–45% of repair. This new approach opens diverse avenues of modifying RNA sequences “à la carte” by exchanging mutated exons, introducing new exons or even correct duplication mutations.

## Results

### Design of trans-splicing molecules

In ExChange molecules, the replacing exon is flanked by artificial intronic sequences with strong acceptor and donor splice sites, which are connected to antisense sequences designed to anneal the target mRNA. Annealing is crucial to permit the trans-splicing reaction, although it is not enough. Ideally, the site of binding must disturb the definition of the targeted exon in the parental pre-messenger while enhancing cross-splicing in between the two independent mRNAs. In the case of ExChange, there are more constraints since two trans-splicing reactions must be synchronized at both edges of the targeted exon.

**Figure 1:**
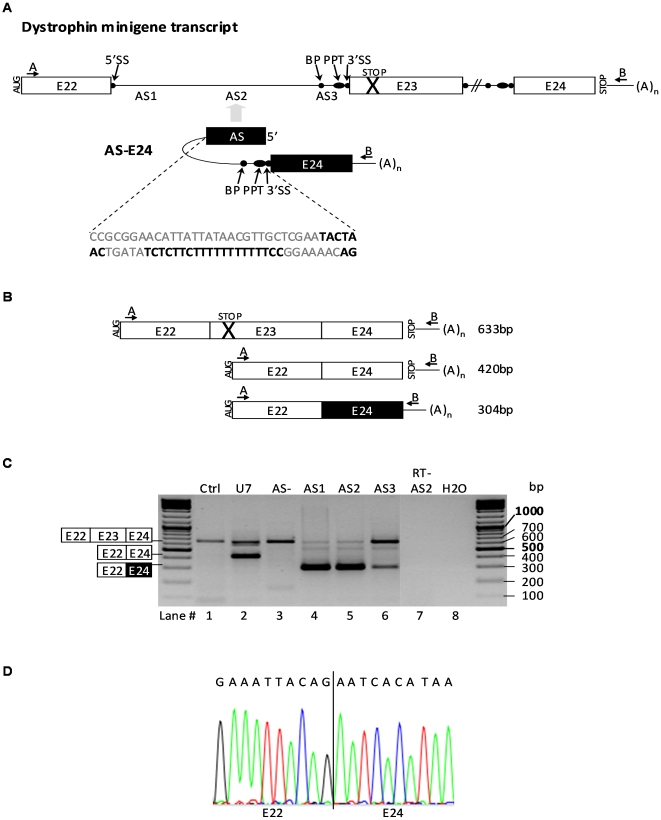
Trans-splicing strategy for 3′ replacement. (A) The murine dystrophin minigene consists of exons 22 to 24 (boxes E22 to E24) with natural introns (lines). The splice sequences are illustrated by black balls: 5′ splice sites (5′SS), branching points (BP), polypyrimidine tracts (PPT) and 3′ acceptor sites (3′SS). The cross represents the nonsense *mdx* mutation in E23. The trans-splicing (TS) molecule AS-E24 comprises a 150 nt antisense sequence (AS) complementary to intron 22 as well as a spacer, a strong conserved yeast branch point sequence, a polypyrimidine tract, a 3′ acceptor site and E24. The sequence of the spacer, BP, PPT and 3′SS is given with the BP, PPT and 3′SS in bold and illustrated by black balls. The E24 sequence from minigene (white box) and TS molecule (black box) are identical, only the downstream sequence differs in length. TS constructs were made with three different antisense sequences, AS1 to AS3. Arrows indicate the positions of the forward A and reverse B PCR primers in the minigene and the TS molecule. (B) Expected transcripts generated by cis-splicing (E23 inclusion and skipping) and trans-splicing, and the predicted sizes of the corresponding PCR amplification products detected using the RT-PCR strategy illustrated in (A). (C) RT-PCR analysis using PCR primers A and B of NIH3T3 cells cotransfected with dystrophin minigene and constructions pSMD2 (Ctrl), pSMD2-U7-SD23-BP22 (U7), pSMD2-E24 (AS-), pSMD2-AS1-E24 (AS1), pSMD2-AS2-E24 (AS2) and pSMD2-AS3-E24 (AS3). RT- AS2: samples containing dystrophin minigene and pSMD2-AS2-E24 without reverse transcription; H2O: PCR negative control. Representative results from three independent transfection experiments. (D) An exact E22-E24 junction was confirmed by sequencing of the 304bp product.

The murine model for dystrophin mutations, the *mdx* mouse, carries a nonsense mutation in exon 23 (E23^m^) of the dystrophin gene. In order to locate the best site of annealing in intron 22, upstream the mutated exon, we designed first three trans-splicing (TS) molecules for 3′ replacement by a single trans-splicing event, only differing in their binding domains (Fig. 1A). Antisense sequences of 150 nucleotides (AS1, AS2 and AS3) were chosen to match either to the 5′ end, the middle or the 3′ end of intron 22. The idea was to test whether getting the TS molecule close to its target, 5′ donor splice site (5′SS) of intron 22, or on the contrary, masking the 3′ acceptor splice site (3′SS), would facilitate trans-splicing. In the three constructions, the artificial intron included a spacer sequence [20], a strong conserved yeast branch point sequence (BP), a polypyrimidine tract (PPT) and a canonical 3′ acceptor splice site (3′SS) [21]. To facilitate the readout of subsequent exon exchange experiments, we also decided to employ exon 24 (E24) in the TS molecule instead of the normal version of exon 23 (E23). Indeed, E24 is smaller than E23 (114 versus 213bp) allowing unequivocal distinction by RT-PCR of repaired mRNA (E22-E24-E24) from non-repaired parental transcripts (E22-E23^m^-E24). As control, we used a trans-splicing molecule with no binding domain (AS-).

To facilitate the analysis of dystrophin splicing in tissue culture, we created a dystrophin minigene made of a murine genomic fragment of 3993bp comprising E22 to E24 with full-length natural introns. Cis- and single trans-splicing patterns are illustrated in Figure 1B. An RT-PCR strategy was designed to detect specifically RNA resulting from cis- and trans-splicing events by using a forward primer E22-F (arrow A in Fig. 1B) specific for E22, and a reverse primer pSMD2-R1 (arrow B) specific for a sequence upstream the polyA signal in the dystrophin minigene and TS molecules. Importantly, these primers also allowed us to discriminate E22-E24 amplicons resulting from either trans-splicing or exon skipping.

### Single trans-splicing approach

Dystrophin minigene and TS plasmids were cotransfected in the mouse embryonic fibroblast NIH3T3 cell-line. Cells were harvested 72 h after transfection, and total RNA was isolated. Cis- and trans-spliced RNA patterns were assessed by RT-PCR. Samples that received only the mutation-bearing dystrophin minigene displayed a single 633bp amplicon corresponding to the cis-spliced transcript E22-E23^m^-E24 (lane 1, Ctrl in Fig. 1C). Also, cDNAs from cells transfected with both dystrophin minigene and trans-splicing constructs (AS2-E24) gave no PCR products when reverse transcription was omitted (lane 7, RT- AS2), ensuring us about the specificity of our assay. In the presence of U7-SD23-BP22 (lane 2, U7) plasmids described to induce E23 skipping [1], a predominant 420bp band corresponding to E22-E24 transcript from cis-splicing was detected.

In samples that received dystrophin minigene and TS plasmids (lanes 4, 5 and 6 in Fig. 1C), a product of 304bp was generated, corresponding specifically to the trans-spliced E22-E24 variant as shown in Figure 1D by the sequence of E22-E24 junction, and not to an exon skipping product as it was obtained with U7. In the presence of either AS1-E24 (lane 4) or AS2-E24 (lane 5), the E22-E23^m^-E24 amplicon corresponding to the parental dystrophin transcript had significantly decreased thus confirming that trans-splicing efficacies were elevated. Efficacies seem so high that the parental template transcript has almost disappeared. We cannot exclude that this unprecedented trans-splicing efficiency was enlarged by a PCR bias benefiting the small trans-spliced amplicons rather than the non-repaired template. As well, total yields (cumulated band intensities of parental and repaired amplicons) are not the same in all lanes, while we used comparable amounts of minigene templates for all transfections. AS1-E24 and AS2-E24 molecules (lanes 4 and 5) were more efficient than AS3-E24 (lane 6), and variations could not be attributed to higher levels of expression of these constructs as shown in Fig. S1B.

Importantly, trans-splicing did not occur when AS was removed (lane 3, AS-) from TS constructs demonstrating that this reaction required close interaction in between the two strands of mRNA to combine. The AS3-E24 TS molecule (lane 6) appeared to be less efficient. Surprisingly, extending AS3 in order to cover the 3′ acceptor site of E23^m^ did not improve the trans-splicing reaction (not shown). These experiments show that trans-splicing could not be induced without AS sequences, although aligning the two mRNAs is not sufficient.

### Double trans-splicing approach: ExChange

**Figure 2:**
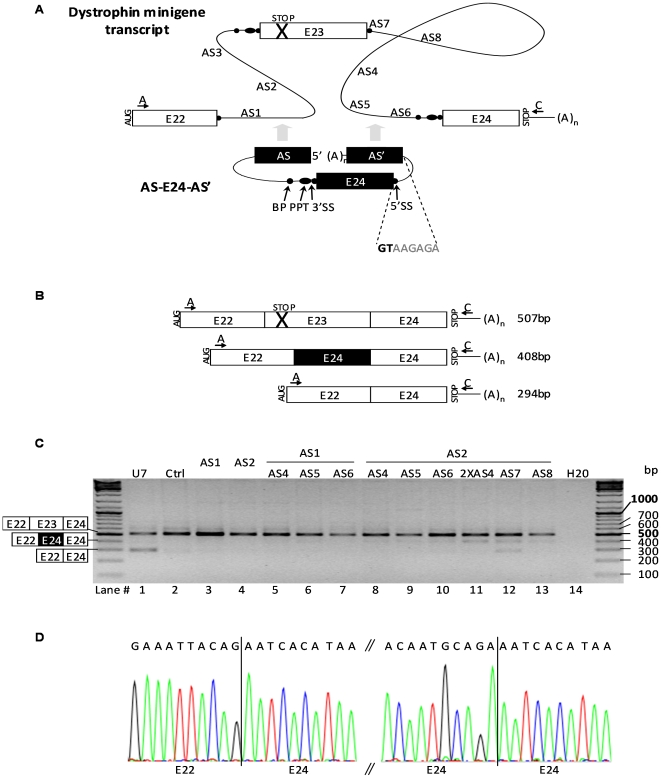
Exon replacement approach on dystrophin minigene transcripts. (A) The exon exchange molecule (ExCh) AS-E24-AS' comprises the same elements as the TS molecule (see Fig. 1A) followed by a 5′ donor splice site (5′SS) and a second antisense sequence (AS') of 150 nt complementary to intron 23. The sequence of 5′SS is given in bold and is illustrated by a black ball. ExCh constructs were made with five different AS' antisense sequences, AS4 to AS8. Arrows indicate the positions of the forward A and reverse C PCR primers in the minigene. (B) Expected transcripts generated by cis-splicing (E23 inclusion and skipping) and exon exchange, and predicted sizes of the corresponding PCR amplification products detected using the RT-PCR strategy illustrated in (A). (C) RT-PCR analysis using primers A and C of NIH3T3 cells cotransfected with dystrophin minigene and constructions pSMD2 (Ctrl), pSMD2-U7-SD23-BP22 (U7), the TS constructions pSMD2-AS1-E24 (AS1), pSMD2-AS2-E24 (AS2) and ExCh molecules pSMD2-AS-E24-AS' containing AS1 or AS2 and AS4 to AS8. AS2-2XAS4, ExCh plasmid pSMD2-AS2-E24-2XAS4 containing two AS4 copies; H20: PCR negative control. Representative results from two independent transfection experiments. (D) Accurate E22-E24 and E24-E24 junctions were confirmed by sequencing of the 408bp product.

To test the possibility of mRNA repair by ExChange, we developed several ExChange (ExCh) molecules AS-E24-AS' based on the efficient TS molecules mentioned above, modified to bind both intron 22 and intron 23 of the dystrophin minigene (Fig. 2A). The ExCh molecules contained the same elements as previously described in AS1-E24 and AS2-E24 TS molecules followed by a 5′ donor splice site (5′SS) [15] and a second 150 nt antisense targeting intron 23. Five antisense sequences, AS5 to AS8, were selected within intron 23, which spans over 2607bp. As described before, ExCh constructs and dystrophin minigene were cotransfected in NIH3T3 cell-line. Cells were harvested 72 h after transfection, and total RNA was extracted. To detect specifically RNA resulting from cis-splicing and/or exon exchange events, we used a forward primer E22-F (arrow A in Fig. 2A–B) specific of E22, and a reverse primer pSMD2-R5 (arrow C) specific of a sequence only present in the dystrophin minigene upstream its polyA signal. Targeting of AS-E24-AS' in the dystrophin minigene pre-mRNA is illustrated in Figure 2A and expected sizes of the various amplification products shown in Figure 2B.

A RT-PCR product of 408bp was detected in samples transfected with AS2-E24-AS' plasmids (lanes 8–13, Fig. 2C). Direct sequencing confirmed that this product corresponded to the exchanged mRNA variant E22-E24-E24 (Fig. 2D). This product was absent when one of the two antisense was lacking (lanes 3 and 4, AS1 and AS2), showing that co-targeting of intron 22 and intron 23 is crucial for ExChange. Among the antisense combinations we tried, levels of the 408bp band were stronger with AS2-E24-2XAS4 (lane 11), -AS7 (lane 12) and -AS8 (lane 13). Interestingly, the AS2-E24-2XAS4 molecule, which carried two AS4, was more efficient than its single AS4 counterpart (lane 5). In AS2-E24-AS7 sample (lane 12), an additional band of 294bp was detected corresponding to E22-E24 transcript generated by exon 23 skipping (see lane 1, U7). This was not surprising considering that AS7 bound the 5′ donor splice site of intron 23 and would mask its recognition by the spliceosome. It is likely that AS4, AS7 and AS8 brought ExCh molecules closer to E23 than AS5 and AS6 suggesting that a tight framing of the target exon is essential for efficient ExChange.

### Optimization of ExChange efficacy by adding up intronic splice enhancers

**Figure 3:**
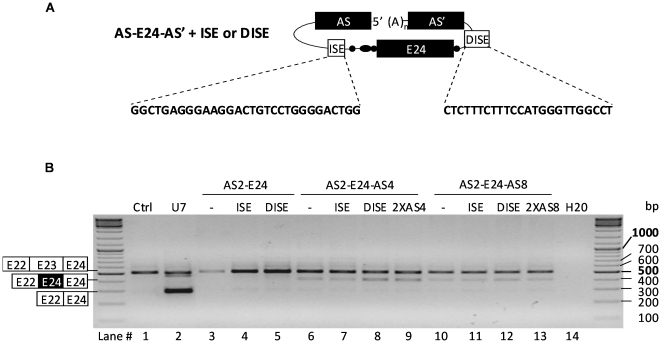
Effect of intronic splice enhancer sequences on exon replacement efficiency. (A) Exon exchange molecules AS-E24-AS' with intronic splice enhancers ISE or DISE sequences. (B) RT-PCR analysis using primers A and C of NIH3T3 cells cotransfected with dystrophin minigene and constructs pSMD2 (Ctrl), pSMD2-U7-SD23-BP22 (U7) and the following ExCh plasmids with AS4 or AS8: pSMD2-AS2-E24-AS' (-), pSMD2-AS2-ISE-E24-AS' (ISE), pSMD2-AS2-E24-DISE-AS' (DISE) and pSMD2-AS2-E24-2XAS' (2XAS'). H20: PCR negative control.

**Figure 4:**
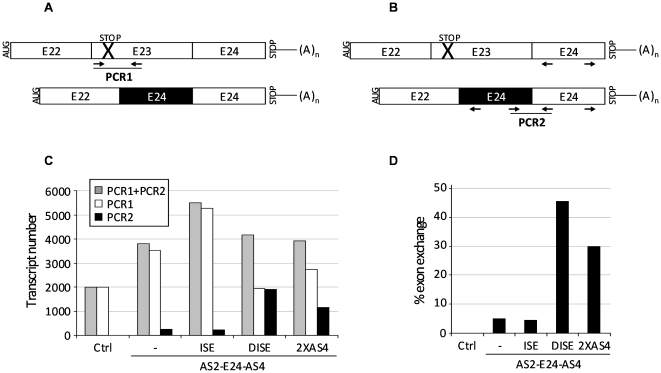
Efficiency of dystrophin exon exchange induced by AS4 containing ExCh molecules. (A) Expected amplicons from PCR1 with E23-F & E23-R primers and (B) from PCR2 with E24-F & E24-R primers. (C) Histogram showing numbers of parental and exchanged transcripts evaluated by absolute quantitative real-time RT-PCR. PCR1 corresponds to the parental transcripts whereas PCR2 to the repaired transcripts. PCR1+PCR2 corresponds to the totality of the dystrophin minigene transcripts. (D) ExChange efficiency estimated by calculating the ratio between levels obtained for repaired transcripts (PCR2) and whole dystrophin minigene transcripts (PCR1+PCR2). Representative results from two independent transfection experiments.

In order to improve the ExChange reaction, the G-rich intronic splice enhancer (ISE) from the human GH-1 gene (GeneID: 2688) was added upstream the 3′ acceptor site of AS2-E24-AS4 and AS2-E24-AS8 [22] and the DISE sequence from the rat FGFR2 gene (GeneID: 25022) downstream the 5′ donor site [14], [23] (Fig. 3A). As shown in Figure 3B, RT-PCR analysis revealed that insertion of the DISE sequence in AS2-E24-AS4 and AS2-E24-AS8 molecules (lanes 8 and 12) increased significantly the 408bp band corresponding to the E22-E24-E24 mRNA variant, while addition of ISE sequence (lanes 7 and 11) did not enhance the ExChange efficacy. As expected, no ExChange was observed with control vectors lacking the downstream AS': AS2-E24, AS2-ISE-E24 and AS2-E24-DISE (lanes 3, 4 and 5). Figure 4 shows the numbers of parental (PCR1) and exchanged (PCR2) transcripts obtained by absolute quantitative RT-PCR (panel C) and corresponding ExChange efficacy of various vectors (panel D) was evaluated by calculating the ratio between levels obtained for repaired transcripts (PCR2) and whole dystrophin minigene transcripts (PCR1+PCR2). The AS2-DISE-E24-AS4 molecule allowed obtaining 45% of exon exchange. Its efficacy was improved by about 9-folds when compared to its counterpart AS2-E24-AS4 lacking the DISE motif. Interestingly, introduction of two redundant downstream AS' (here AS4) improved ExChange efficacy, which was about 30%. However, AS2-E24-DISE-2XAS4 was not more efficient than AS2-DISE-E24-AS4 (data not shown). Accordingly to the results obtained with AS2-E24-AS8 (lanes 10–13 in Fig. S2A), it is likely that highest efficacy of DISE and 2XAS4 did not resulted from transcriptional over activity of subsequent constructs.

**Figure 5:**
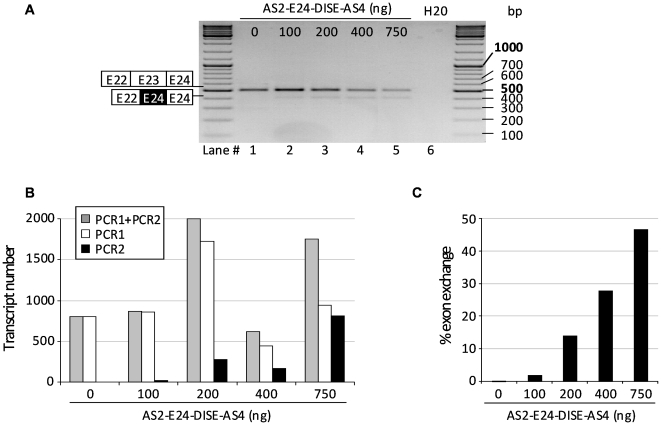
Dosing study of AS2-E24-DISE-AS4 molecule. (A) RT-PCR analysis using primers A and C of NIH3T3 cells cotransfected with 250 ng of dystrophin minigene and 100, 200, 400 and 750 ng of pSMD2-AS2-E24-DISE-AS4 plasmids. (B) Histogram showing numbers of parental and exchanged transcripts evaluated by absolute quantitative real-time RT-PCR illustrated in Fig. 4A. PCR1 corresponds to the parental transcripts whereas PCR2 to the repaired transcripts. PCR1+PCR2 corresponds to the totality of the dystrophin minigene transcripts. (C) ExChange efficiency of the dosing study estimated by calculating the ratio between levels obtained for repaired transcripts (PCR2) and whole dystrophin minigene transcripts (PCR1+PCR2). Representative results from two independent transfection experiments.

We next performed a dosing study of the best ExCh molecule AS2-E24-DISE-AS4 in the cotransfection experiments (Fig. 5), maintaining constant the amount of dystrophin minigene while increasing the amount of the ExCh molecule. Although we used comparable amounts of minigene plasmid in all transfections, absolute quantitative RT-PCR on subsequent samples showed some discrepancies in transcript levels as shown in Figure 5B (PCR1+PCR2). Therefore, analysis on agarose gel was only used to confirm the absence of unexpected PCR products (Fig. 5A), while ExChange efficacy was evaluated by absolute quantitative RT-PCR by calculating the ratio between levels obtained for repaired transcripts (PCR2) and whole dystrophin minigene transcripts (PCR1+PCR2) (Fig. 5C). ExChange efficiency improved with the amount of ExCh molecule in a quasi linear manner to reach 50% of repair with one dystrophin minigene (250 ng) for three ExCh molecules (750 ng) encoding plasmids.

### Exon 23 repair by exon replacement

**Figure 6:**
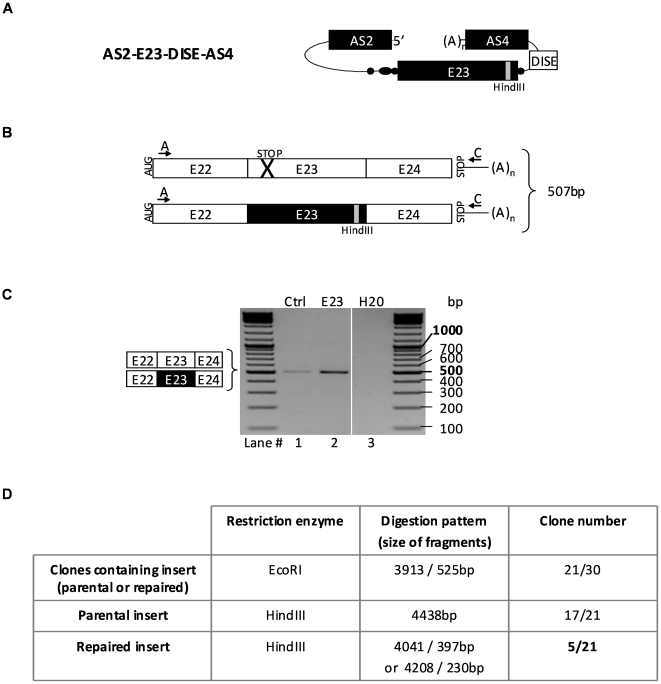
Exon 23 correction by exon replacement. (A) The ExCh AS2-E23-DISE-AS4 molecule comprises the same elements as AS2-E24-DISE-AS4, except E23 instead of E24. E23 is the wild-type murine dystrophin E23 carrying a HindIII restriction site absent in the *mdx* minigene E23. (B) Expected transcripts generated by cis-splicing and E23 exchange, and predicted size of the corresponding RT-PCR products using primers A and C. (C) RT-PCR analysis using primers A and C of NIH3T3 cells cotransfected with dystrophin minigene and constructions pSMD2 (Ctrl) and ExCh plasmid pSMD2-AS2-E23-DISE-AS4; H20: PCR negative control. Representative results from two independent transfection experiments. (D) Cloning results of the RT-PCR amplicons obtained in (C) with AS2-E23-DISE-AS4 molecule. The clone numbers corresponding to parental and repaired inserts are described.

Having identified the best antisense targets within introns 22 and 23 of the dystrophin gene, and customized flanking acceptor and donor splice sites of the ExChange molecule, we settled on a new ExChange molecule designed to replace the mutated *mdx* exon 23 by its wild-type version (Fig. 6A). Since both parental and repaired minigene transcripts gave rise to similar RT-PCR products (507bp) with primer E22-F and pSMD2-R5 (arrows A and C in Fig. 6B), we included a HindIII restriction site into the wild type E23. The 507bp PCR-product obtained in samples transfected with AS2-E23-DISE-AS4 plasmid (lane 2 in Fig. 6C, E23) was cloned into pCR®-2.1-TOPO® plasmid. Thirty clones were obtained which were analyzed by EcoRI restriction for the presence of E22-E23-E24 insert and by HindIII restriction to substantiate the repaired amplicon. Among these 30 clones, 21 contained an E22-E23-E24 insert. Among them, 5 displayed the HindIII site, the attribute of the repaired dystrophin minigene product, meaning that about 25% of the minigene transcripts were repaired (Fig. 6D and Fig. S3). Wild-type exon 23 exchange in these clones was confirmed by direct sequencing.

## Discussion

This study demonstrates the possibility of rescuing mutated transcripts by specifically replacing the mutated exon by its normal version during the splicing reaction. We relate here the optimization and add-ons to classical trans-splicing molecules (3′ replacement) required for efficient exon exchange (ExChange: concomitant 3′ and 5′ trans-splicing reactions). To achieve this goal, we set up an experimental model to detect both repaired and non-repaired transcripts in the same detection system, allowing accurate estimation of mRNA repair. The ExChange efficiency reached a level of 45% (when using exon 24 as ExChange reporter) and 25% (when using wild type exon 23) of repaired transcripts with the ExCh molecule containing the DISE motive in our cotransfection assay with dystrophin minigene as target.

In order to show that the repair occurred at mRNA level by double trans-splicing and not by plasmid recombination, two independent mutations were included in the pSMD2-AS2-E24-DISE-AS4 construct. First, the CMV promoter was deleted to prevent expression of the AS2-E24-DISE-AS4 ExCh molecule. Second, the 5′ splice site GT of the AS2-E24-DISE-AS4 molecule was mutated to CT to create the splicing-deficient ExChange molecule AS2-E24-DISE-AS4-Δ5′SS. Absence of significant plasmid recombination was also checked after DNA recovery from cotransfected cells with dystrophin minigene and pSMD2-AS2-E24-DISE-AS4. In the three conditions, we were not able to detect any band corresponding to the repaired minigene product by RT-PCR (Fig. S4), thus confirming that ExChange indeed required a fully functional ExChange mRNA molecule, and did not occur at the level of plasmid-DNA recombination.

Importantly, we never found abnormal or non-expected end products, suggesting that chosen annealing sequences did not bind to cryptic splicing sites nor obstruct slicing events in the target mRNA. This is likely due to the use of long antisense sequences (150 nt) [24], which were not chosen in the definition motives of targeted exons. In actual fact it is impossible to state that there is no non-specific trans-splicing event. In our experiments, removing one of the two antisense domains disabled ExChange demonstrating that annealing with the target was crucial to achieve detectable trans-splicing reactions. Still, one might hypothesize that “off targets” might trigger unexpected trans-splicing reactions in the presence of the antisense domains in the final construct. Finally, we have carried out the classical experiment to assess non-specific trans-splicing by using co-transfection with an irrelevant target gene, in this case an ACTA1 exogenous gene. No non-specific trans-splicing events were detected (data not shown).

The trans-splicing technology uses a trans-splicing molecule that “tricks” the spliceosome into using it as a substrate for splicing. In the ExChange approach, the game is more “tricky” since the spliceosome must carry out a double trans-splicing between the pre-messenger transcript and the ExCh molecule. After having tested various combinations of antisense sequences, we were surprised to realize that the dogmas: i) blocking endogenous splicing signals on the nascent pre-mRNA transcript via base-pairing or ii) on the contrary, bringing the replacing exon closer to endogenous splice site to be joined, did not produce the best results. Our best antisense for the first trans-splicing (3′ replacement) matched with the middle of the first intron while the second one (5′ replacement) was better when located close to the 3′ end of the exon to be replaced. Postulating that length of the ExCh molecule is critical for its efficiency, would explain the necessity of a close framing of the exon to be replaced. Moreover, the spacers are certainly important elements of the ExChange molecule, and it may be of interest to study the influence of their length and composition on ExChange efficacy. In the case of 5′ replacement, the spacer element can be removed without decreasing efficacy of trans-splicing constructs [15]. Conversely, in 3′ replacement, the spacer sequence is crucial since it contains intronic consensus sequences required for the recruitment of auxiliary factors involved in the acceptor site recognition and exon processing. For ExChange, ideally, both 5′ and 3′ reactions need to be synchronized, thus requiring that the two antisense sequences concomitantly anneal the same target mRNA molecule. In this case, it is likely that the spacer length must fit conformational constraints dictated by the distance between the two antisense target sequences. On the other hand, ExChange could also arise by means of two independent target mRNAs, which would be involved sequentially. This requires retaining the first trans-spliced intermediate in the nucleus, so that the second reaction might take place with a different mRNA target. A manner to retain the first intermediate in the splicing compartment could be achieved by introducing intronic elements which could bind elements of the splicing machinery, such as DISE motives, thus signaling that splicing is not yet achieved. In such a view, length and composition of the spacers might be crucial for the persistence of the intermediate ExChange mRNA chimera.

As a RNA repair strategy, the ExChange approach will produce the corrected protein where it is naturally expressed. It has the further advantage over other RNA surgery strategies of correcting precisely the sequence defect without altering the rest of the messenger sequence (i.e. the open reading frame and untranslated regions). Hence, the regulatory sequences present in 5′ and 3′ UTRs are preserved, something which never happens in classical gene therapy where cDNAs are amputated of their non-coding sequences. These regions are now known to be essential for mRNA stability and translation regulation; in particular, they are targets for miRNAs which play important role in a variety of diseases [25]. Furthermore, ExChange strategy may allow for multiple isoforms to be repaired. In particular, this new technology could allow therapeutic solution for genes as complex as the dystrophin gene with a variety of mutations and multiple isoforms.

To conclude, tools and rules provided by this study open new avenues in the field of investigation of trans-splicing. In addition, the ExChange strategy allows a sort of surgery of the mRNA molecule for therapeutic purpose (genetic rescue) as well as for basic research (protein chimeras).

## Materials and Methods

### Plasmids constructions

The murine dystrophin minigene target (3993bp) comprising exons E22, E23, and E24 and the natural E23 flanking intronic sequences was constructed by PCR amplification from *mdx* genomic DNA and subcloned in pSMD2 [26] into KpnI site. An ATG in Kozak ACCACCATGG context and a STOP codon were introduced at both sides of the minigene. For the TS and ExCh molecules, E24 (114bp) was amplified from the dystrophin minigene whereas E23 (213bp) from normal murine dystrophin cDNA. The E23 lysine codon (nucleotide +196, where nucleotide +1 is the first E23 nucleotide) was transformed from AAA to AAG creating a HindIII restriction site. The different domains of the TS and ExCh molecules detailed in Results section were constructed by PCR and subcloned in pSMD2 into HindIII and EcoRI between the CMV promoter and the polyA signal. Antisense sequences AS bind to dystrophin intron 22: AS1 targets nucleotides −763 to −614; AS2, −463 to −314; AS3 −159 to −10; AS3bis −159 to +5 (where nucleotide +1 is the first E23 nucleotide). The second antisense domains AS' bind to dystrophin intron 23: AS4, +1801 to +1950; AS5, +2101 to +2250; AS6, +2401 to +2550; AS7, −5 to +145; AS8, +151 to +300 (where nucleotide +1 is the first nucleotide of intron 23). All expression cassettes are under the control of the strong CMV promoter and a polyA signal. The pool of E22-E23-E24 parental and repaired amplicons were cloned into the pCR®-2.1-TOPO®. All the constructs were verified by sequencing.

### Cell culture and transfection

Mouse embryonic fibroblast NIH3T3 cells (ATCC) were maintained in DMEM (Invitrogen) supplemented with 10% heat-inactivated FBS (Invitrogen), 100 units/ml penicillin, and 100 µg/ml streptomycin. For transfections, cells were grown to 70% confluence in 12-well plates and exposed to the DNA/Lipofectamine 2000 reagent (Invitrogen) complex for 5 h in DMEM before being returned to normal culture medium. Typically, 250 ng of dystrophin minigene and 750 ng of TS or ExCh molecules DNA were used in each transfection except for the dosing study where 250 ng of dystrophin minigene were cotransfected with 100, 200, 400 and 750 ng of pSMD2-AS2-E24-DISE-AS4 completed with pSMD2 plasmid to reach a total amount of 1 µg per point. Cells were routinely analyzed 72 h after transfection.

### RT-PCR analysis

Total RNA was isolated from transfected cells by using RNAeasy extraction kit (Qiagen). Reverse transcription was performed on 200 ng of RNA by using the Superscript II (Invitrogen) and the reverse primer pSMD2-R1 (see below) at 10 min at 25°C, 50 min at 42°C, and a final step of 5 min at 95°C. To detect non-repaired and repaired dystrophin transcripts, reverse transcribed RNA was amplified by PCR under the following conditions: 95°C for 5 min, 30 cycles of 30 s at 95°C, 1 min at 56°C, 45 s to 1 min at 72°C, and a final step of 7 min at 72°C.

The sequences of the primers were as followed: E22-F GACACTTTACCACCAATGCGC (Primer A on Fig. 1A–B and 2A–B), pSMD2-R1 CTTTCTGATAGGCAGCCTGC (Primer B on Fig. 1A–B) and pSMD2-R5 CTCACCCTGAAGTTCTCAGG (Primer C on Fig. 2A–B). RT-PCR products were separated by electrophoresis in 2% agarose gels with ethidium bromide and sequenced.

### Quantitative real-time RT-PCR

mRNA levels were measured by absolute quantitative real-time RT-PCR method using Absolute SYBR Green Rox Mix (Thermo scientific). Two positive control dystrophin cDNA fragments, E22-E23-E24 and E22-E24-E24, were cloned into the pCR®-2.1-TOPO®. As a reference samples, those plasmids were 10-fold serially diluted (from 10^7^ to 10^3^ copies) and used to generate standard curves. Real-time PCR was performed and analyzed on a DNA Engine Opticon 2 (Bio-Rad). In each experiment, duplicates of standard dilution series of control plasmids and first strand cDNA generated by the Superscript II (Invitrogen) and the reverse primer pSMD2-R1 from 200 ng of total RNA were amplified by specific primers. Primers for E23, E23-F AGATGGCCAAGAAAGCACC and E23-R CTTTCCACCAACTGGGAGG, were used to measure non-repaired dystrophin transcript; and primers for E24-E24 junction, E24-F TGAAAAAACAGCTCAAACAATGC and E24-R AGCATCCCCCAGGGCAGGC, for the repaired transcript. ExChange efficiency was estimated by calculating the ratio between levels obtained for repaired transcripts (E24-F & E24-R) and whole dystrophin minigene transcripts, approximated by summing levels of non-repaired transcripts (E23-F & E23-R) plus the repaired subset (E24-F & E24-R).

## Supporting Information

Figure S1 Expression levels of ExChange molecules. Expression levels of ExChange molecules were estimated by subtracting the number of minigene transcripts, estimated by quantitative PCR with A/D primers (E22-F & E22-R) to the number of E24 copies, estimated by quantitative PCR E/F primers (E24-F2 & E24-R2): ExCh copy number = (E/F)−(A/D). mRNA levels were measured by absolute quantitative real-time RT-PCR methods as described previously using pCRÂ®-2.1-TOPOÂ®-E22-E23-E24 as reference sample and primers E22-F, E22-R CCGAGTCTCTCCTCCATTATTTC, E24-F2 CACATAAAAACCTTACAGAAATG and E24-R2 CTGCATTGTTTGAGCTGTTTTTTC. (A) Expected transcripts and the anticipated PCR amplifications with A/D and E/F primers. (B) Trans-splicing strategy for 3′ replacement. RT-PCR analysis using PCR primers A and B of NIH3T3 cells cotransfected with dystrophin minigene and constructions pSMD2 (Ctrl), pSMD2-U7-SD23-BP22 (U7), pSMD2-E24 (AS-), pSMD2-AS1-E24 (AS1), pSMD2-AS2-E24 (AS2) and pSMD2-AS3-E24 (AS3). RT- AS2: samples containing dystrophin minigene and pSMD2-AS2-E24 without reverse transcription; H2O: PCR negative control. Histogram shows the number of TS molecules expressed for 100 copies of dystrophin minigene transcripts. AS1-E24 and AS2-E24 molecules (lanes 4 and 5) are the most efficient. AS3-E24 construct (lane 6) was highly expressed but promoted lower levels of trans-splicing. (C) Exon replacement approach on dystrophin minigene transcripts. RT-PCR analysis using primers A and C of NIH3T3 cells cotransfected with dystrophin minigene and constructions pSMD2 (Ctrl), pSMD2-U7-SD23-BP22 (U7), the TS constructions pSMD2-AS1-E24 (AS1), pSMD2-AS2-E24 (AS2) and ExCh molecules pSMD2-AS-E24-AS' containing AS1 or AS2 and AS4 to AS8. AS2-2XAS4, ExCh plasmid pSMD2-AS2-E24-2XAS4 containing two AS4 copies; H20: PCR negative control. Histogram shows the number of ExCh molecules expressed for 100 copies of dystrophin minigene transcripts. AS2-E24-2XAS4 construct (lane 11) is not highly expressed but is the most efficient molecule in the ExChange experiments.
(5.95 MB TIF)

Figure S2 Expression levels of ExChange molecules. Expression levels of ExChange molecules were estimated by subtracting the number of minigene transcripts, estimated by quantitative PCR with A/D primers (E22-F & E22-R) to the number of E24 copies, estimated by quantitative PCR E/F primers (E24-F2 & E24-R2): ExCh copy number = (E/F)−(A/D). mRNA levels were measured by absolute quantitative real-time RT-PCR methods as described previously using pCRÂ®2.1-TOPOÂ®-E22-E23-E24 as reference sample and primers E22-F, E22-R CCGAGTCTCTCCTCCATTATTTC, E24-F2 CACATAAAAACCTTACAGAAATG and E24-R2 CTGCATTGTTTGAGCTGTTTTTTC. (A) Effect of intronic splice enhancer sequences on exon replacement efficiency. RT-PCR analysis using primers A and C of NIH3T3 cells cotransfected with dystrophin minigene and constructs pSMD2 (Ctrl), pSMD2-U7-SD23-BP22 (U7) and the following ExCh plasmids with AS4 or AS8: pSMD2-AS2-E24-AS' (−), pSMD2-AS2-ISE-E24-AS' (ISE), pSMD2-AS2-E24-DISE-AS' (DISE) and pSMD2-AS2-E24-2XAS' (2XAS'). H20: PCR negative control. Histogram shows the number of ExCh molecules expressed for 100 copies of dystrophin minigene transcripts. DISE and 2XAS4 constructs are the most efficient (lanes 8 and 9). Accordingly to the results obtained with AS2-E24-AS8 (lanes 10–13), it is clear that highest efficacy of DISE and 2XAS4 did not resulted from transcriptional over activity of these constructs. (B) Dosing study of AS2-E24-DISE-AS4 molecule. RT-PCR analysis using primers A and C of NIH3T3 cells cotransfected with 250 ng of dystrophin minigene and 100, 200, 400 and 750 ng (lanes 2–5) of pSMD2-AS2-E24-DISE-AS4 plasmids. Histogram shows the number of ExCh molecules expressed for 100 copies of dystrophin minigene transcripts and confirms that ExChange efficacy increases accordingly to the amounts of AS2-E24-DISE-AS4 molecules.
(3.66 MB TIF)

Figure S3 Restriction analysis of clones containing E22-E23-E24 inserts. The 507bp PCR-product obtained in samples transfected with AS2-E23-DISE-AS4 plasmid (Fig. 6C) was cloned into pCRÂ®-2.1-TOPOÂ® plasmid. Thirty clones were obtained which were analyzed by EcoRI restriction for the presence of E22-E23-E24 insert and by HindIII restriction to substantiate the repaired amplicon. Among these 30 clones, 21 contained an E22-E23-E24 insert (discernible by *). Among them, 5 displayed the HindIII site (*), the feature of the repaired dystrophin minigene product. Wild type exon 23 exchange in these clones was confirmed by direct sequencing.
(9.64 MB TIF)

Figure S4 Control experiments to exclude contribution of plasmid recombination. In order to show that the repair occurred at mRNA level by double trans-splicing and not by plasmid recombination, two independent mutations were included in the pSMD2-AS2-E24-DISE-AS4 construct. First, the CMV promoter was deleted to prevent expression of the AS2-E24-DISE-AS4 ExCh molecule, pSMD2-AS2-E24-DISE-AS4-delCMV. Second, the 5′ splice site GT of the AS2-E24-DISE-AS4 molecule was mutated to CT to create the splicing-deficient ExChange molecule AS2-E24-DISE-AS4-del5′SS. Absence of significant plasmid recombination was also checked after DNA recovery from cotransfected cells with dystrophin minigene and pSMD2-AS2-E24-DISE-AS4 (DNA). After RT-PCR with primers A and C (see Fig. 2B), no 408bp band corresponding to the repaired minigene product was detected in the three conditions (lanes 3–5, delCMV, del5′SS and DNA), thus confirming that previously documented ExChange (lane 2) did not occur at the level of plasmid recombination and required a fully functional ExChange molecule.
(5.77 MB TIF)
