# Supplementary figures and images for: Exon Exchange Approach to Repair Duchenne Dystrophin Transcripts
Source: PLoS One. 2010 May 28;5(5):e10894. doi: 10.1371/journal.pone.0010894 (PMC2878348; doi:10.1371/journal.pone.0010894)

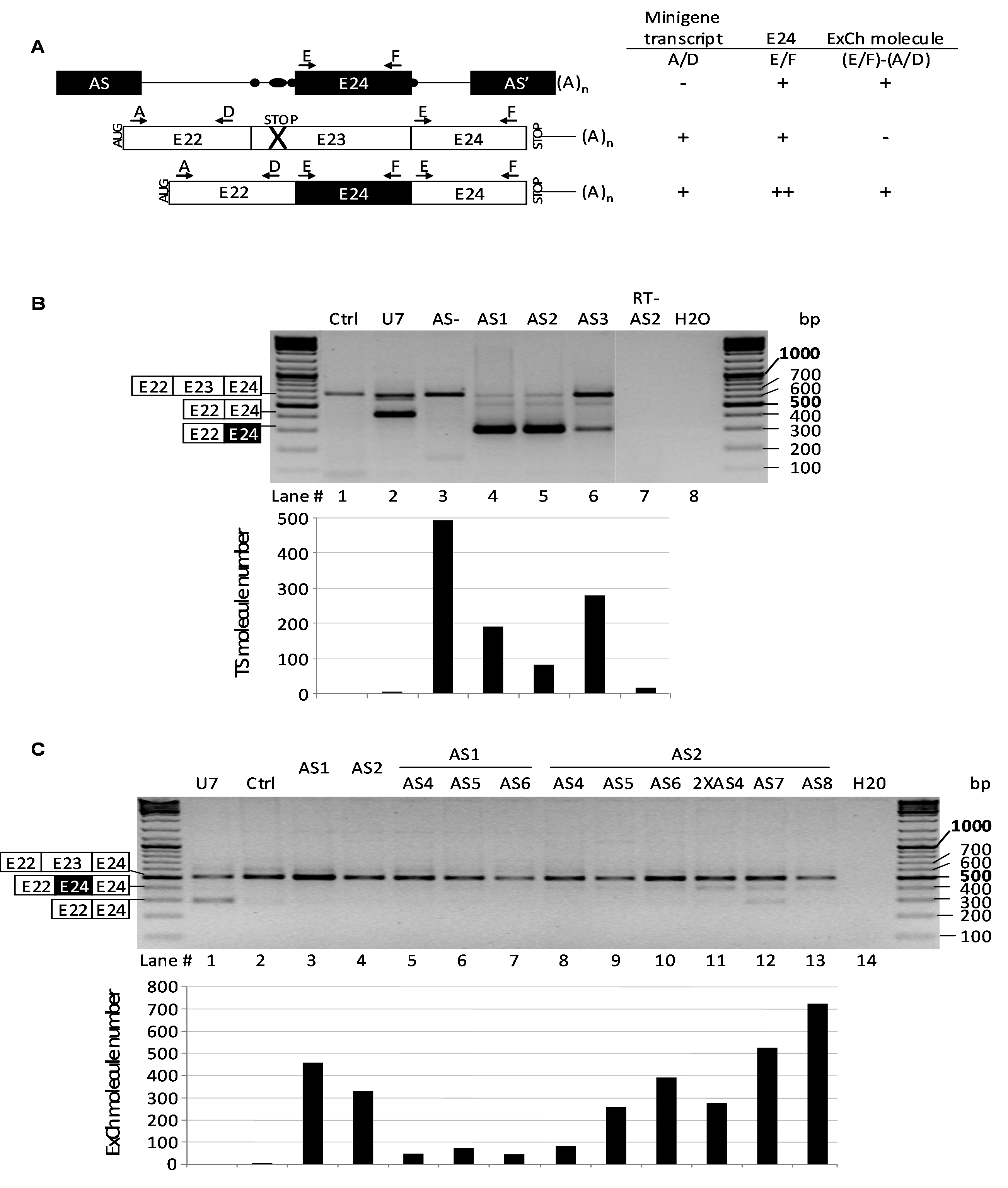

Supplement: Figure S1 — Expression levels of ExChange molecules. Expression levels of ExChange molecules were estimated by subtracting the number of minigene transcripts, estimated by quantitative PCR with A/D primers (E22-F & E22-R) to the number of E24 copies, estimated by quantitative PCR E/F primers (E24-F2 & E24-R2): ExCh copy number = (E/F)−(A/D). mRNA levels were measured by absolute quantitative real-time RT-PCR methods as described previously using pCRÂ®-2.1-TOPOÂ®-E22-E23-E24 as reference sample and primers E22-F, E22-R CCGAGTCTCTCCTCCATTATTTC, E24-F2 CACATAAAAACCTTACAGAAATG and E24-R2 CTGCATTGTTTGAGCTGTTTTTTC. (A) Expected transcripts and the anticipated PCR amplifications with A/D and E/F primers. (B) Trans-splicing strategy for 3′ replacement. RT-PCR analysis using PCR primers A and B of NIH3T3 cells cotransfected with dystrophin minigene and constructions pSMD2 (Ctrl), pSMD2-U7-SD23-BP22 (U7), pSMD2-E24 (AS-), pSMD2-AS1-E24 (AS1), pSMD2-AS2-E24 (AS2) and pSMD2-AS3-E24 (AS3). RT- AS2: samples containing dystrophin minigene and pSMD2-AS2-E24 without reverse transcription; H2O: PCR negative control. Histogram shows the number of TS molecules expressed for 100 copies of dystrophin minigene transcripts. AS1-E24 and AS2-E24 molecules (lanes 4 and 5) are the most efficient. AS3-E24 construct (lane 6) was highly expressed but promoted lower levels of trans-splicing. (C) Exon replacement approach on dystrophin minigene transcripts. RT-PCR analysis using primers A and C of NIH3T3 cells cotransfected with dystrophin minigene and constructions pSMD2 (Ctrl), pSMD2-U7-SD23-BP22 (U7), the TS constructions pSMD2-AS1-E24 (AS1), pSMD2-AS2-E24 (AS2) and ExCh molecules pSMD2-AS-E24-AS' containing AS1 or AS2 and AS4 to AS8. AS2-2XAS4, ExCh plasmid pSMD2-AS2-E24-2XAS4 containing two AS4 copies; H20: PCR negative control. Histogram shows the number of ExCh molecules expressed for 100 copies of dystrophin minigene transcripts. AS2-E24-2XAS4 construct (lane 11) is not highly expressed but is the most [file pone.0010894.s001.tif]

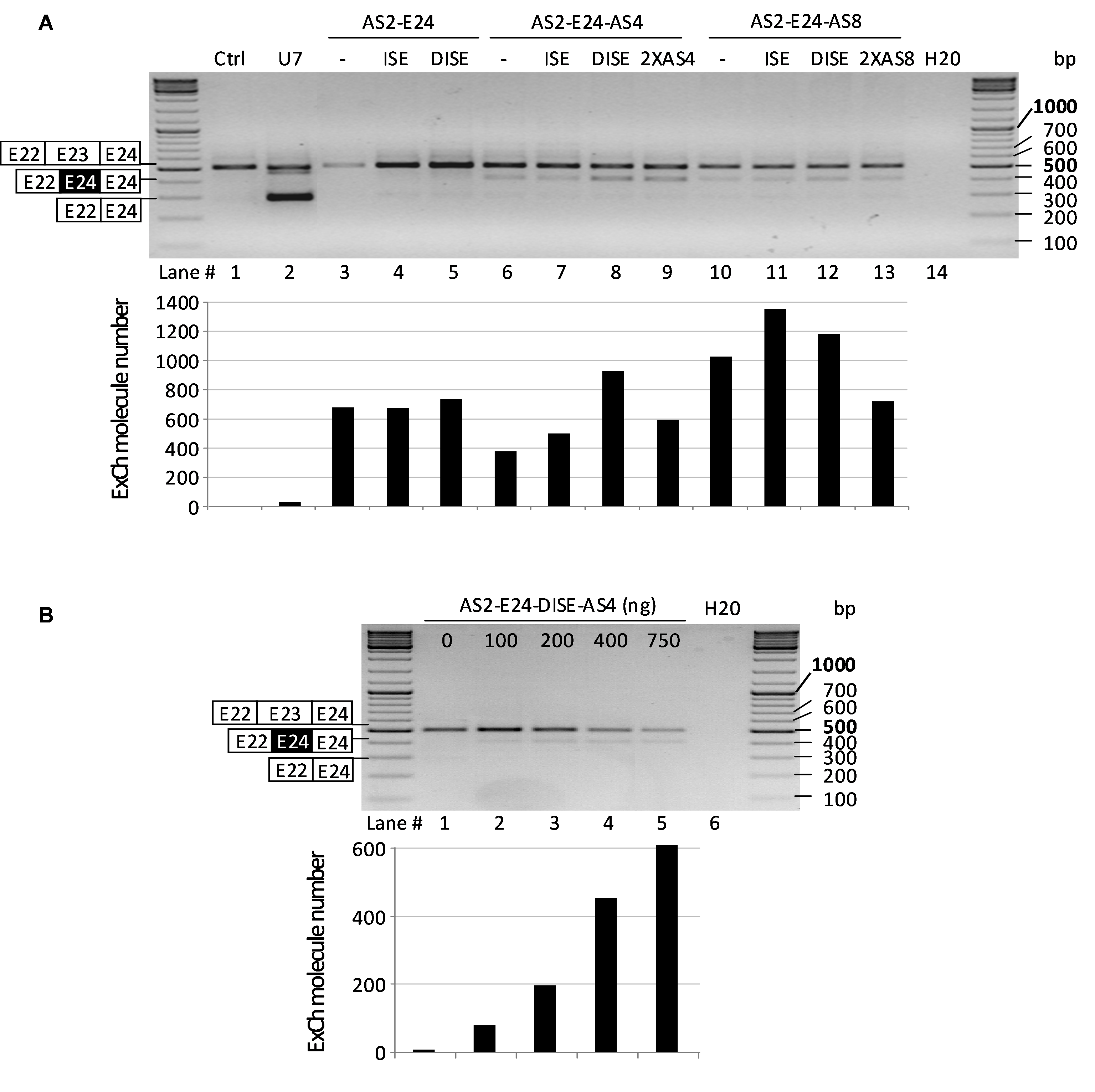

Supplement: Figure S2 — Expression levels of ExChange molecules. Expression levels of ExChange molecules were estimated by subtracting the number of minigene transcripts, estimated by quantitative PCR with A/D primers (E22-F & E22-R) to the number of E24 copies, estimated by quantitative PCR E/F primers (E24-F2 & E24-R2): ExCh copy number = (E/F)−(A/D). mRNA levels were measured by absolute quantitative real-time RT-PCR methods as described previously using pCRÂ®2.1-TOPOÂ®-E22-E23-E24 as reference sample and primers E22-F, E22-R CCGAGTCTCTCCTCCATTATTTC, E24-F2 CACATAAAAACCTTACAGAAATG and E24-R2 CTGCATTGTTTGAGCTGTTTTTTC. (A) Effect of intronic splice enhancer sequences on exon replacement efficiency. RT-PCR analysis using primers A and C of NIH3T3 cells cotransfected with dystrophin minigene and constructs pSMD2 (Ctrl), pSMD2-U7-SD23-BP22 (U7) and the following ExCh plasmids with AS4 or AS8: pSMD2-AS2-E24-AS' (−), pSMD2-AS2-ISE-E24-AS' (ISE), pSMD2-AS2-E24-DISE-AS' (DISE) and pSMD2-AS2-E24-2XAS' (2XAS'). H20: PCR negative control. Histogram shows the number of ExCh molecules expressed for 100 copies of dystrophin minigene transcripts. DISE and 2XAS4 constructs are the most efficient (lanes 8 and 9). Accordingly to the results obtained with AS2-E24-AS8 (lanes 10–13), it is clear that highest efficacy of DISE and 2XAS4 did not resulted from transcriptional over activity of these constructs. (B) Dosing study of AS2-E24-DISE-AS4 molecule. RT-PCR analysis using primers A and C of NIH3T3 cells cotransfected with 250 ng of dystrophin minigene and 100, 200, 400 and 750 ng (lanes 2–5) of pSMD2-AS2-E24-DISE-AS4 plasmids. Histogram shows the number of ExCh molecules expressed for 100 copies of dystrophin minigene transcripts and confirms that ExChange efficacy increases accordingly to the amounts of AS2-E24-DISE-AS4 molecules. (3.66 MB TIF) [file pone.0010894.s002.tif]

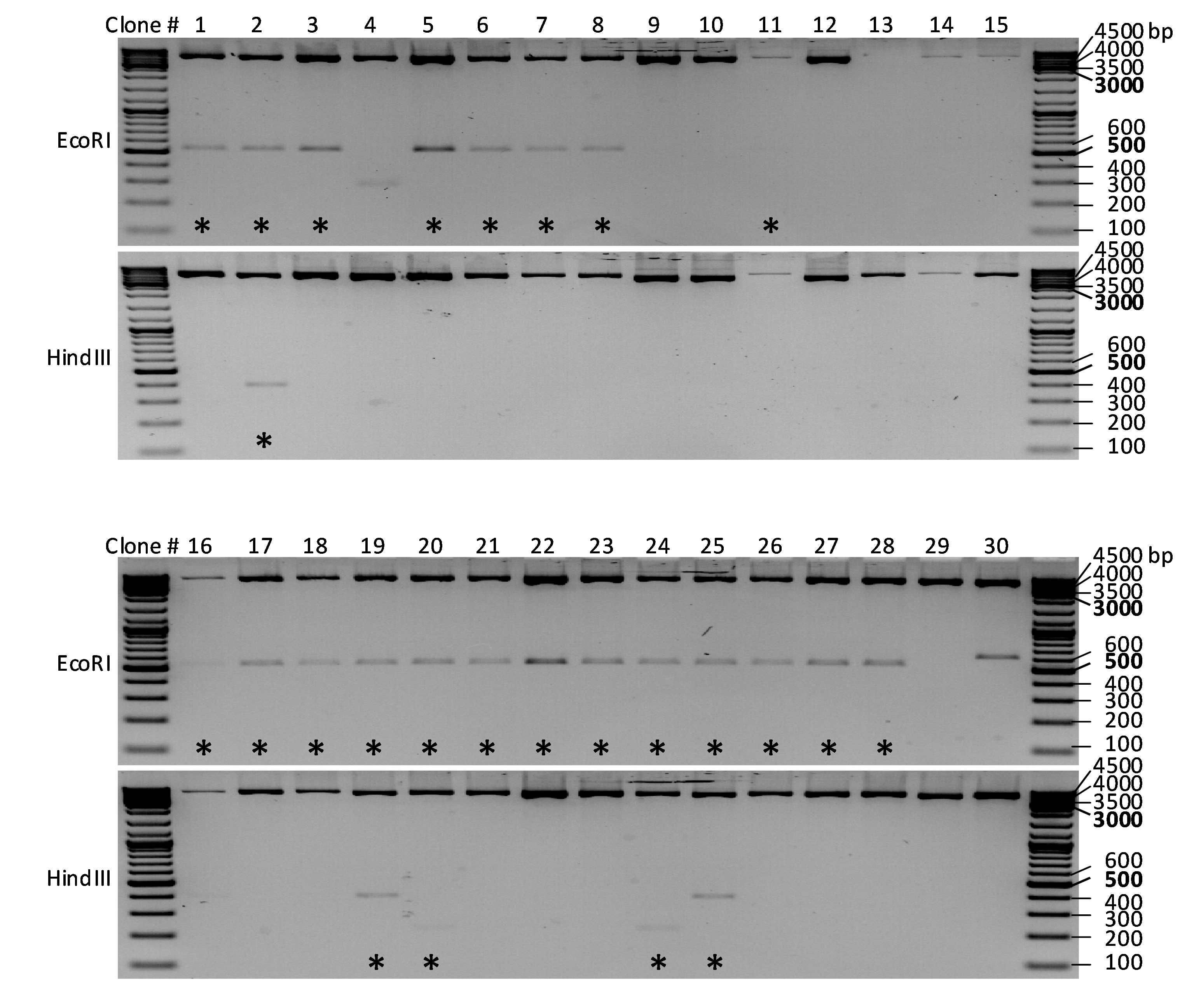

Supplement: Figure S3 — Restriction analysis of clones containing E22-E23-E24 inserts. The 507bp PCR-product obtained in samples transfected with AS2-E23-DISE-AS4 plasmid (Fig. 6C) was cloned into pCRÂ®-2.1-TOPOÂ® plasmid. Thirty clones were obtained which were analyzed by EcoRI restriction for the presence of E22-E23-E24 insert and by HindIII restriction to substantiate the repaired amplicon. Among these 30 clones, 21 contained an E22-E23-E24 insert (discernible by *). Among them, 5 displayed the HindIII site (*), the feature of the repaired dystrophin minigene product. Wild type exon 23 exchange in these clones was confirmed by direct sequencing. (9.64 MB TIF) [file pone.0010894.s003.tif]

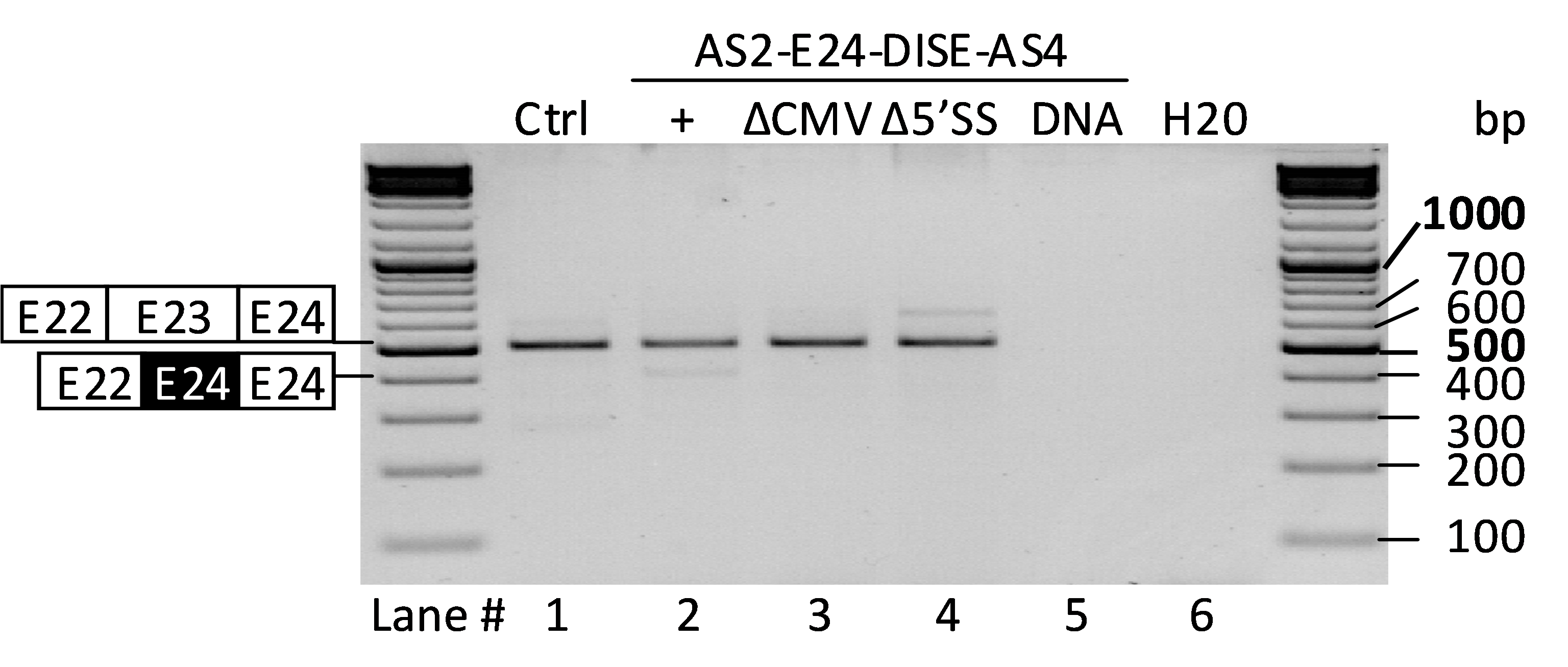

Supplement: Figure S4 — Control experiments to exclude contribution of plasmid recombination. In order to show that the repair occurred at mRNA level by double trans-splicing and not by plasmid recombination, two independent mutations were included in the pSMD2-AS2-E24-DISE-AS4 construct. First, the CMV promoter was deleted to prevent expression of the AS2-E24-DISE-AS4 ExCh molecule, pSMD2-AS2-E24-DISE-AS4-delCMV. Second, the 5′ splice site GT of the AS2-E24-DISE-AS4 molecule was mutated to CT to create the splicing-deficient ExChange molecule AS2-E24-DISE-AS4-del5′SS. Absence of significant plasmid recombination was also checked after DNA recovery from cotransfected cells with dystrophin minigene and pSMD2-AS2-E24-DISE-AS4 (DNA). After RT-PCR with primers A and C (see Fig. 2B), no 408bp band corresponding to the repaired minigene product was detected in the three conditions (lanes 3–5, delCMV, del5′SS and DNA), thus confirming that previously documented ExChange (lane 2) did not occur at the level of plasmid recombination and required a fully functional ExChange molecule. (5.77 MB TIF) [file pone.0010894.s004.tif]
